# Prognostic Value of Different Iron Status Definitions in Congestive Heart Failure: A Retrospective MIMIC-IV Analysis of Risk Stratification and Mortality

**DOI:** 10.3390/jcm15010244

**Published:** 2025-12-28

**Authors:** Abdulla Zahi Hourani, Arman David Sürmeli, Sai Keertana Devarapalli

**Affiliations:** 1 Faculty of Medicine, English Division, Medical University of Warsaw, 02-091 Warsaw, Poland; armansuermeli@gmail.com (A.D.S.);; 2 Department and Clinic of Nephrology, Dialysis and Internal Medicine, Medical University of Warsaw, 02-091 Warsaw, Poland

**Keywords:** iron deficiency, heart failure, transferrin saturation, ferritin, MIMIC-IV, mortality

## Abstract

**Background**: Iron deficiency (ID) is prevalent in congestive heart failure (CHF), worsening outcomes. While European guidelines recommend screening using ferritin and transferrin-saturation (TSAT), inconsistent diagnostic criteria, especially regarding functional deficiency (ferritin 100–299 μg/L + TSAT < 20%) and hyperferritinemia, limit prognostic accuracy. This study evaluated iron status definitions, including guideline criteria and a combined Ferritin-TSAT model, for predicting 365-day mortality in hospitalised CHF patients. **Methods**: This retrospective analysis used MIMIC-IV data from 1839 CHF patients. Iron status within 24 h of admission was categorised using: (1) Guideline ID vs. non-ID; (2) Ferritin categories; (3) TSAT categories; (4) Combined Ferritin-TSAT model (Low: guideline ID; Intermediate: ferritin 100–299 + TSAT ≥ 20%; High: ferritin ≥ 300 μg/L). Adjusted Cox models assessed mortality associations. **Results**: Guidelines-defined iron deficiency (33.66% prevalence) independently associated with higher 1-year mortality (56.1% vs. 29.4%; adjusted HR 4.36, 95% CI 3.35–5.34). The combined Ferritin-TSAT model showed significant prognostic value, differentiating true iron deficiency (reference) from hyperferritinemia (adjusted HR 0.50 vs. iron deficiency) and intermediate group (adjusted HR 0.36 vs. ID), indicating varying risk relative to the most deficient group. This combined model better distinguished hyperferritinemic and iron-replete subgroups than the binary guideline definition. **Conclusions**: Iron status, including deficiency and hyperferritinemia, independently predicts 1-year mortality in CHF. While guideline iron deficiency is a strong predictor, a combined Ferritin-TSAT classification offers finer risk stratification by identifying distinct phenotypes (true deficiency, hyperferritinemia, intermediate). Nuanced iron status assessment could improve prognostic evaluation and guide personalised therapies (e.g., IV iron for deficiency, investigation for hyperferritinemia) to enhance CHF outcomes.

## 1. Introduction

Iron deficiency anaemia is a significant global public health concern and affects a substantial proportion of patients with congestive heart failure (CHF), with estimates suggesting that as many as 34–63% of individuals with chronic heart failure may be iron-deficient [1]. Iron deficiency itself can adversely influence outcomes, by contributing to poor exercise tolerance, diminished health-related quality of life, and increased risks of hospitalisation and mortality, even in the absence of anaemia [2,3]. Recognising the central role of iron deficiency in exacerbating congestive heart failure symptoms, the European Society of Cardiology has formally acknowledged iron deficiency as an important comorbidity and now recommends routine testing of iron status for all newly diagnosed heart failure patients [4,5].

Iron deficiency in CHF patients can manifest as either absolute iron deficiency (depleted iron stores) or functional iron deficiency (adequate iron stores that cannot be effectively mobilised) [6,7]. This creates a state where iron is unavailable for critical cellular processes despite being present in the body [8,9]. The prevailing consensus holds that iron deficiency, irrespective of anaemia, directly contributes to heart failure pathophysiology by impairing mitochondrial energy production, contributing to maladaptive cardiac remodelling, and activating inflammatory pathways that collectively reduce cardiac output [10].

Multiple large-scale trials and meta-analyses have consistently highlighted the clinical benefits of correcting iron deficiency in CHF. In particular, intravenous (IV) iron supplementation has been shown to alleviate symptoms, improve exercise capacity, and reduce heart failure–related hospitalisations [4,11,12,13,14]. Observational and randomised evidence further indicate that these advantages extend beyond anaemic patients to those with normal haemoglobin levels but low TSAT (<20%), which nonetheless signals depleted functional iron stores. This reflects the necessity of targeting iron deficiency, guided by iron parameters like TSAT and ferritin, as a central therapeutic strategy in heart failure, rather than relying on haemoglobin levels to dictate treatment decisions.

However, the clinical utility of these findings is hindered by inconsistent diagnostic criteria, as there remains no universally accepted definition of iron imbalance in the forms of depletion or excess in CHF for mortality prediction. Cutoff values for ferritin and TSAT in the definition of iron deficiency vary among studies, with recent studies advocating for sole TSAT oriented operational definitions [15,16], creating a lack of standardisation that complicates the analysis of iron deficiency prevalence [1]. According to the most recent recommendations published by the European Society of Cardiology and their focused update, iron deficiency in heart failure is defined as a serum ferritin concentration < 100 μg/L, or 100–299 μg/L with a transferrin saturation (TSAT) < 20%, corresponding to absolute and functional iron deficiency, respectively [4,17,18]. Despite ESC guidance, the prognostic validity of these thresholds for long-term, post-discharge mortality remains unclear. Beyond classical deficiency, hyperferritinemia (≥300 μg/L), often reflecting inflammation or congestion, may be associated with a distinct risk profile; differentiating this phenotype from true deficiency and normal iron status may improve prognostic classification and motivate phenotype-specific evaluation.

In light of the above, this study focuses on further investigating the effects and optimal diagnostic criteria in prognosis of iron deficiency in congestive heart failure. By examining the impact of iron deficiency on long-term mortality beyond the acute hospital setting, this research aims to address the ongoing need for clearer, evidence-based guidelines on screening and treating iron deficiency in CHF. To this end, the study rigorously evaluates and compares different stratification approaches, including a refined combined model, to overcome limitations in current definitions.

## 2. Materials and Methods

### 2.1. Study Design and Setting

We conducted a retrospective observational cohort study of adult hospitalisations with CHF using routinely collected electronic health record (EHR) data from a large tertiary academic medical centre in the United States. The analysis was performed on the de-identified Medical Information Mart for Intensive Care IV (MIMIC-IV) database, which integrates information from intensive care units and affiliated hospital wards within Beth Israel Deaconess Medical Centre (2008–2019) [19]. The database contains detailed longitudinal data, including demographics, admission and discharge information, vital signs, laboratory measurements, procedures, diagnostic and procedure codes, medication information, and free-text clinical documentation for each hospitalisation.

For this study, all eligible CHF inpatient stays recorded in the hospital module over the predefined study period were screened at the admission level and subsequently linked using unique patient identifiers to construct a cohort of index hospitalisations. Structured EHR tables (e.g., admissions, diagnoses, procedures, ICU stays, and laboratory results) were merged with unstructured clinical notes.

### 2.2. Patient Selection and Data Extraction

Inclusion criteria were ages between >18 and <89 years old, validated ICD-9/10 diagnosis of CHF, availability of admission notes, and iron parameters obtained within 24 h of admission; only the first hospitalisation per patient was retained. Patients with a length of stay < 2 days were excluded, consistent with other studies. Patients aged ≥89 years were also excluded due to MIMIC age top-coding for data compliance, as this group is statistically rare and potentially re-identifiable.

Clinical notes were analysed using a Gemma2-9B-Instruct-based extraction pipeline similar to the EchoLLM framework [20], but adapted to focus exclusively on left ventricular ejection fraction (LVEF). We prompted the model, with ten hand-crafted few-shot example echocardiography snippets, to return a structured JSON field containing LVEF as a numeric value (collapsing reported ranges to the most abnormal bound) and, when only qualitative language was present, to map LVEF into guideline-defined categories (reduced, mildly reduced, preserved); few-shot prompting was used to enhance extraction accuracy [21,22]. Targeted regular-expression post-processing was applied to capture any remaining numeric EF mentions and to cross-check CHF diagnoses. A random subset of 100 notes was independently labelled by two authors (A.H. and A.S.) for LVEF category (reduced, mildly reduced, preserved) based on the source text, with no disagreements on this subset (100% agreement; weighted Cohen’s κ = 1.00). The extraction pipeline was then compared against these manual labels. Performance is reported as one-vs-rest sensitivity (recall) and specificity for each class, together with macro-F1. The pipeline achieved macro-F1 = 0.92; per-class sensitivity/specificity were: reduced [Se 0.93, Sp 0.98], mildly reduced (Se 0.87, Sp 0.99), and preserved (Se 0.89, Sp 0.95).

Demographic and clinical variables (e.g., age, sex, comorbidities including shock, malignancy, renal disease, as well as ICU stay and ejection fraction status) were collected from structured EMR fields and supplemented by natural language processing. Laboratory measurements for ferritin, iron, TIBC, TSAT, haemoglobin, and other routine labs (e.g., creatinine for eGFR calculation) were obtained from the first tests within 24 h of admission. Diagnoses were established via ICD-9/10 codes corroborated by notes. EF categories were used for phenotypic stratification and covariate adjustment. CKD was defined by ICD-9/10 codes for chronic renal insufficiency; AKI by ICD-9/10 codes consistent with KDIGO criteria. Procedures were identified using ICD procedure codes. Mineralocorticoid receptor antagonists (MRAs), angiotensin receptor–neprilysin inhibitors (ARNIs), and sodium–glucose cotransporter-2 inhibitors (SGLT2i) were not uniformly available or adopted in clinical practice during the study period. These therapies were not included in the present analysis.

### 2.3. Definition of Iron Deficiency

Iron deficiency was defined using guideline-defined thresholds of ferritin < 100 μg/L or ferritin 100–299 μg/L with TSAT < 20% [17]; non-iron deficiency otherwise. Analyses evaluated iron deficiency irrespective of anaemia status; haemoglobin was included as an adjusted covariate and summarised at baseline.

### 2.4. Data Cleaning and Categorisation

All continuous variables were subjected to thorough assessment of distributional assumptions, including the Anderson–Darling test (for deviations in skewness and kurtosis), the Kolmogorov–Smirnov test (for overall distributional fit), and visual evaluation using histograms and Q–Q plots. Little’s test did not reject the hypothesis that data were missing completely at random (MCAR), and the observed missingness pattern was consistent with MCAR. Consequently, we proceeded with complete-case analysis.

Patients were categorised based on guideline-defined iron deficiency versus non-iron deficiency, clinically defined ferritin category, TSAT category, and a combined Ferritin-TSAT category classification. Given this study’s focus on mortality prediction, evaluating the prognostic utility of iron deficiency definitions was prioritised over distinguishing between functional and absolute subtypes, as clinical guidelines and prior evidence emphasise iron parameters’ collective role in risk stratification [9]. Category separation for the tested models was based on current guidelines and clinical thresholds. The guideline-defined model classified patients as iron-deficient (ferritin < 100 μg/L or ferritin 100–299 μg/L with transferrin saturation (TSAT) < 20%) versus non-iron-deficient. The Ferritin Category Model defined ferritin < 100 μg/L as deficient, 100–299 μg/L as intermediate, and ≥300 μg/L as hyperferritinemic. In the TSAT Category Model, TSAT < 10% was considered very low, 10–20% low, and ≥20% normal [23]. The combined Ferritin-TSAT Category Model defined low iron status as ferritin < 100 μg/L or 100–299 μg/L with TSAT < 20%, iron-replete as ferritin 100–299 μg/L with TSAT ≥ 20%, and hyperferritinemic as ≥300 μg/L. These classification frameworks were used to explore how variations in iron parameters relate to clinical outcomes while considering iron deficiency and overload. Importantly, the rationale for using the ferritin < 100 μg/L and ferritin 100–299 μg/L with TSAT < 20% cutoffs in the combined model was not only guideline alignment, but also external validity: these thresholds mirror the operational definitions used to enrol patients in major randomised HF intravenous iron trials enabling direct comparison of prognostic strata with trial-tested populations.

### 2.5. Statistical Analyses

Statistical analyses were conducted to compare differences across guideline-defined iron deficiency groups, with the choice of parametric (*t*-tests, ANOVA) or nonparametric (Wilcoxon, Kruskal–Wallis) tests determined by distributional assessments. Categorical variables were compared using chi-square tests or Fisher’s exact test, depending on expected cell counts. Summary statistics for continuous variables were reported as means with standard deviations (SD) for normally distributed data or medians with interquartile ranges (IQR) for skewed distributions, while categorical variables were expressed as frequencies and percentages.

For time-to-event analysis, Kaplan–Meier survival curves were generated to visualise differences in 365-day mortality across study groups. Follow-up started at index admission and continued to 365 days, while only mortality was captured beyond 2019. Log-rank tests were performed to assess statistical significance between survival curves. To further quantify the association between iron parameters and mortality, Cox proportional hazards models were employed to estimate hazard ratios (HRs) with 95% confidence intervals (CIs). The proportional hazards assumption was evaluated using Schoenfeld residuals.

To account for potential confounders, three progressively adjusted Cox models were constructed for each classification framework. Model 1 adjusted for age, sex, ICU admission, and ejection fraction category, accounting for basic demographic and clinical severity factors. Model 2 built upon Model 1 by additionally adjusting for cardiac arrest, hypertension, diabetes, shock, malignancy, haemoglobin, and estimated glomerular filtration rate (eGFR) to incorporate comorbid conditions and laboratory markers relevant to both iron metabolism and cardiovascular outcomes. Model 3 further adjusted for dialysis, percutaneous coronary intervention (PCI) or coronary artery bypass grafting (CABG), beta blockers, angiotensin-converting enzyme inhibitors (ACEi) or angiotensin receptor blockers (ARBs), and iron supplementation, allowing for a comprehensive evaluation of treatment-related influences on mortality.

Subgroup analyses were performed on categories of: age ≥ 65 vs. <65, sex, and reduced vs. mildly-reduced vs. preserved ejection fraction to assess additional adjustments for potential confounding variables. Statistical significance was set at *p* < 0.05, and all analyses were performed using R (version 4.3).

### 2.6. Ethical Considerations

This study was conducted in accordance with the principles of the Declaration of Helsinki and relevant institutional regulations for research using human data. All analyses were performed on the de-identified MIMIC-IV v2.2 database, which was created with approval from the institutional review boards of Beth Israel Deaconess Medical Centre and the Massachusetts Institute of Technology, with a waiver of individual informed consent due to the use of de-identified data. Investigators completed the required training and data use agreements prior to accessing the database. Because the present work is a secondary analysis of an existing de-identified dataset, it did not involve any direct patient contact, did not affect clinical care, and posed minimal risk to participants. No attempt was made to re-identify individuals, and all data handling and reporting followed best practices to preserve patient privacy and confidentiality.

## 3. Results

A total of 1839 unique index admissions, with confirmed congestive heart failure, fulfilled all prespecified eligibility criteria; of which, 1220 had no guideline-defined iron deficiency, while 619 did meet the criteria. Marked imbalances in baseline characteristics were observed between iron-deficient and non-iron-deficient patients, and are summarised in Table 1. Compared with non-iron-deficient patients, iron-deficient patients were older by around 5 years (median 77 vs. 72) and more often female (+14.3 percentage points). Laboratory contrasts included markedly lower ferritin (median 71.0 vs. 392.50 μg/L), iron (31.00 vs. 45.00 μg/dL) and TSAT (9.6% vs. 19.9%), but higher TIBC and transferrin. Clinically, iron-deficient patients had fewer admissions for surgical interventions, shorter hospital stays and lower prevalence of shock (14.5% vs. 25.3%), acute kidney injury (14.2% vs. 25.8%), and myocardial infarction (14.2% vs. 23.4%), yet higher rates of atrial fibrillation (53.3% vs. 45.8%) and valvopathies (49.1% vs. 35.6%). Iron-deficient patients had higher anticoagulant therapy (51.4% vs. 40.1%) but lower vasopressor use (20.8% vs. 36.1%).

Within this cohort, guideline-defined iron deficiency was present in 33.7% (N = 619), leaving 66.3% categorised as non-iron-deficient. Application of the 3-tiered combined Ferritin-TSAT Category Model onto the same cohort reclassified patients into true iron deficiency (33.7%; ferritin < 100 μg/L or ferritin 100–299 μg/L with TSAT < 20%), hyperferritinemia (40.4%; ferritin ≥ 300 μg/L), and iron-replete (25.9%; ferritin 100–299 μg/L with TSAT ≥ 20%) groups, subdividing the non-iron-deficient group into iron-replete and hyperferritinemic phenotypes while retaining the same iron-deficient definition. Overall 365-day all-cause mortality was 38.4% (N = 706) with survival diverging sharply by iron status. Guideline-defined iron deficiency was associated with an absolute 26.7 percentage-point higher 1-year mortality (56.1% vs. 29.4%).

**Figure 1:**
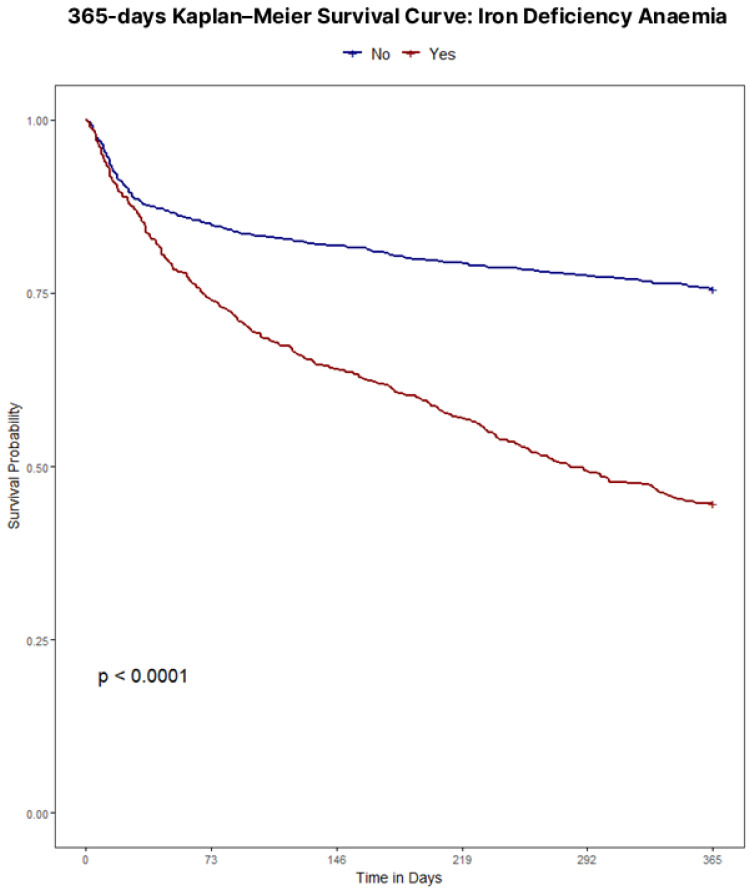
Kaplan–Meier survival to 365 days based on current guideline definitions. Population: hospitalised CHF index admissions. Groups: iron deficiency (ID) (ferritin < 100 μg/L or ferritin 100–299 μg/L with transferrin saturation (TSAT) < 20%) vs. non-ID. Time zero: admission. Outcome: all-cause mortality within 365 days. Deaths were ascertained from linked death records and counted within 365 days of admission, including events occurring after 2019; non-mortality follow-up was restricted to the dataset timeframe. Statistics: log-rank test with numbers at risk shown.

**Figure 2:**
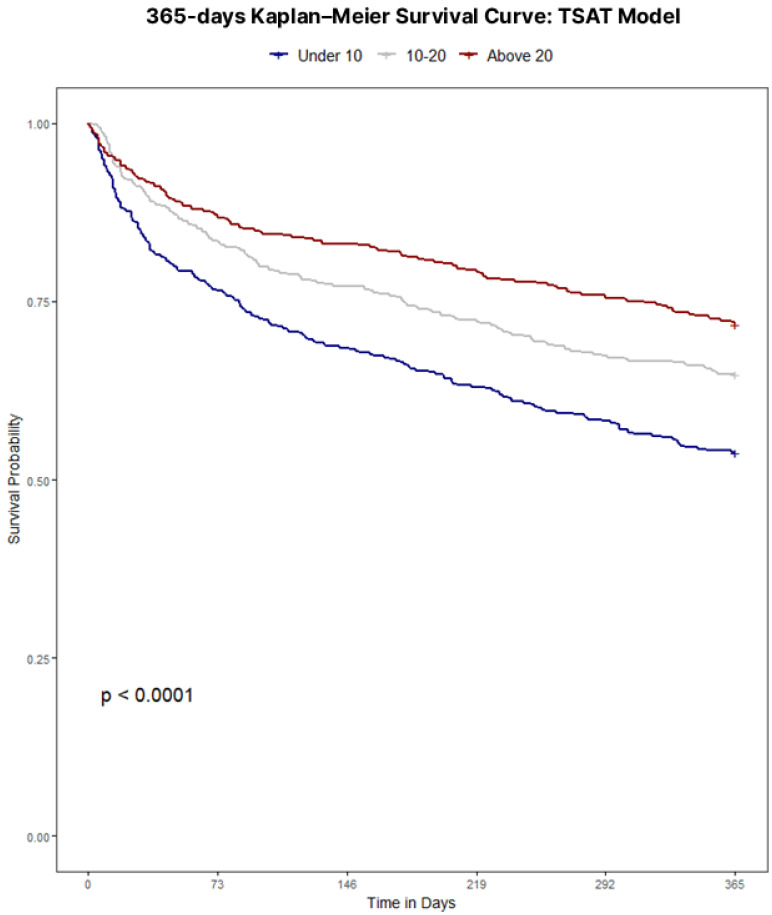
Kaplan–Meier survival to 365 days based on a TSAT category model. Population: hospitalised CHF index admissions. Groups: TSAT < 10% (very low), TSAT 10–20% (low), TSAT ≥ 20% (normal). Time zero: admission. Outcome: all-cause mortality to day 365. Deaths were ascertained from linked death records and counted within 365 days of admission, including events occurring after 2019; non-mortality follow-up was restricted to the dataset timeframe. Statistics: log-rank test with numbers at risk shown. Abbreviations: TSAT, transferrin saturation.

**Figure 3:**
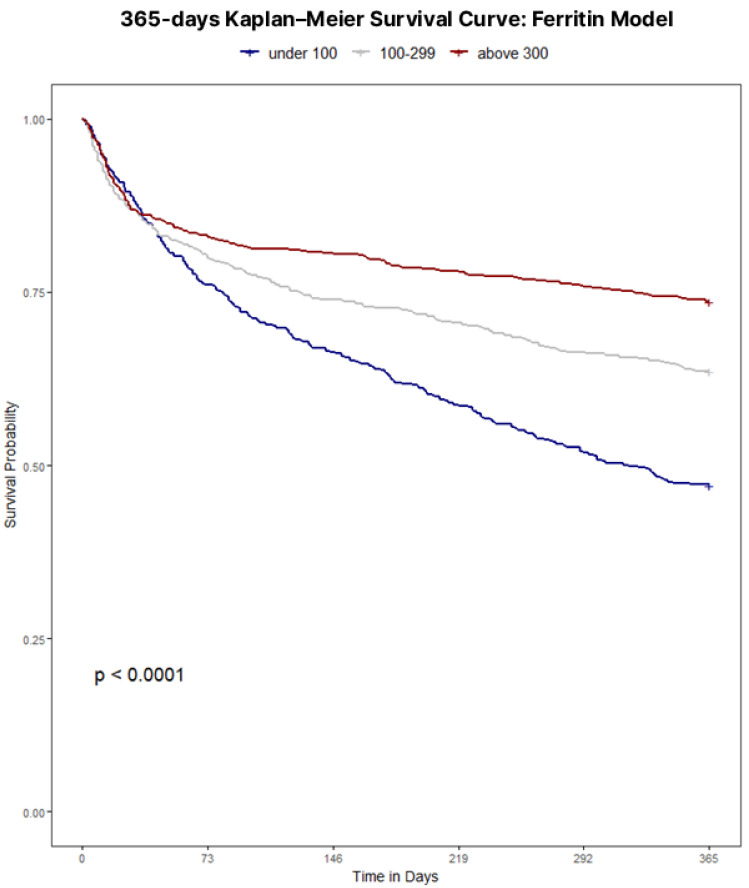
Kaplan–Meier survival to 365 days based on a Ferritin category model. Population: hospitalised CHF index admissions. Groups: ferritin < 100 μg/L (low), 100–299 μg/L (intermediate), ≥300 μg/L (hyperferritinemia). Time zero: admission. Outcome: all-cause mortality to day 365. Deaths were ascertained from linked death records and counted within 365 days of admission, including events occurring after 2019; non-mortality follow-up was restricted to the dataset timeframe. Statistics: log-rank test with numbers at risk shown.

**Figure 4:**
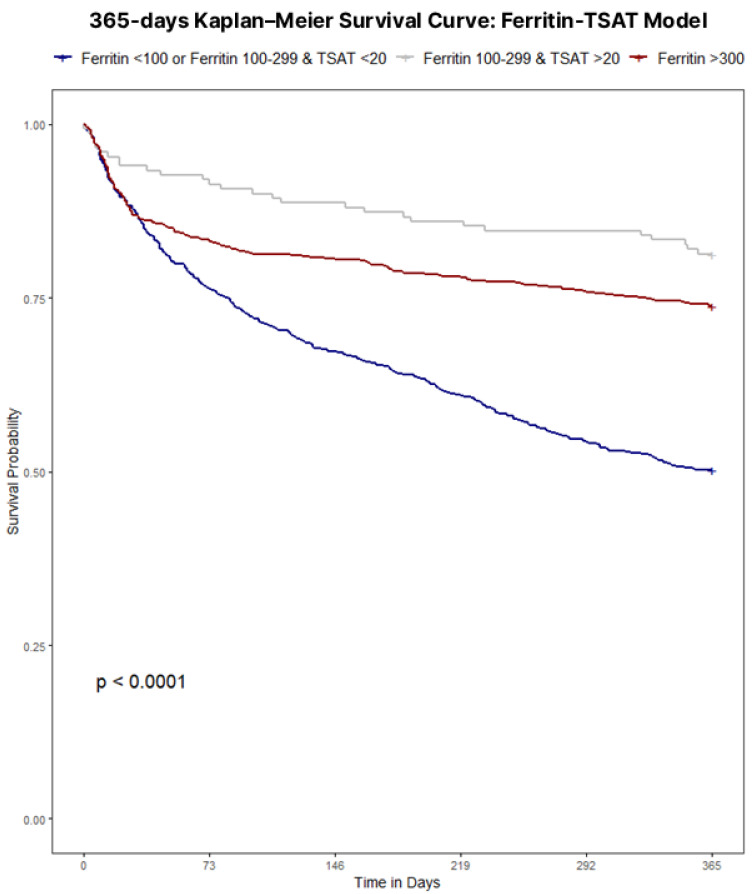
Kaplan–Meier survival to 365 days based on a combined Ferritin-TSAT category model. Population: hospitalised CHF index admissions. Groups: Deficient (ferritin < 100 μg/L or 100–299 μg/L with TSAT < 20%), Hyperferritinemia (ferritin ≥ 300 μg/L), Intermediate (ferritin 100–299 μg/L with TSAT ≥ 20%). Time zero: admission. Outcome: all-cause mortality to day 365. Deaths were ascertained from linked death records and counted within 365 days of admission, including events occurring after 2019; non-mortality follow-up was restricted to the dataset timeframe. Statistics: log-rank test with numbers at risk shown. Interpretive note: early and sustained curve separation is consistent with distinct deficiency vs. inflammation/congestion phenotypes. Abbreviations: TSAT, transferrin saturation.

Throughout the four definition models, iron status retained a clear, independent association with 365-day mortality. Unadjusted survival analyses confirmed poorer outcomes with iron deficiency regardless of definition, with Kaplan–Meier curves illustrating survival for each definition displayed in Figure 1, Figure 2, Figure 3 and Figure 4. Univariate analyses confirmed a strong crude, more than double mortality hazard association between guideline-defined iron deficiency and 365-day mortality (HR 2.48, 95% CI 2.09–2.94). In the TSAT Category Model (reference: TSAT < 10%), TSAT 10–20% and TSAT ≥ 20% were associated with lower hazard (HR 0.72, 95% CI 0.61–0.85; and HR 0.58, 95% CI 0.42–0.78, respectively). In the Ferritin Category Model (reference: ferritin < 100 μg/L), ferritin 100–299 μg/L and ferritin ≥ 300 μg/L were associated with lower hazard (HR 0.66, 95% CI 0.51–0.81; and HR 0.39, 95% CI 0.30–0.57, respectively). In the combined Ferritin-TSAT Category Model, the iron-replete and hyperferritinemia groups were also associated with lower hazard (HR 0.31, 95% CI 0.23–0.50; and HR 0.52, 95% CI 0.32–0.58, respectively) (Table 2). After sequential, fully adjusted multivariate regression modelling for demographics, comorbidity and treatment, including age, sex, ICU stay, shock, malignancy, diabetes, renal function, haemoglobin, dialysis status, invasive procedures, and guideline-directed therapies (full list of covariates available in Appendix A Table A1, Table A2, Table A3 and Table A4), these trends continued, as the guideline-defined model remained independently associated with a 4.36-fold higher hazard of 365-day mortality (95% CI 3.35–5.34) in the fully adjusted model, but lacked the granularity as seen in the Ferritin-TSAT category model, isolating hyperferritinemic and iron-replete phenotypes from the iron-deficient reference with HR 0.50 (95% CI 0.38–0.51) and HR 0.36 (95% CI 0.24–0.53) respectively. To note, in these category models, HR < 1 indicates a lower mortality hazard (lower hazard of death) relative to the reference group, not absolute treatment benefit. Across frameworks, higher age, ICU stay, shock, diabetes and malignancy were associated with increased 365-day mortality, whereas CABG, ACEi/ARB therapy, beta-blockers use and intravenous iron were each associated with lower hazard.

Model discrimination was satisfactory and subgroup analyses confirmed stable performance with minimal variation in hazard estimates within demographic and clinical strata (Table 3). The choice of 65 years as an age cutoff during subgroup analysis aligned with standard practice in cardiology research.

## 4. Discussion

This study shows that, in this retrospective cohort, iron dysregulation in CHF—spanning deficiency to hyperferritinemia—was associated with one-year survival and functioned as a prognostic marker. By evaluating novel categorisations of iron status, we move beyond binary classifications to identify distinct risk phenotypes (biochemical subgroups). Guideline-defined iron deficiency remained a strong, clinically interpretable predictor of 365-day mortality in fully adjusted models, whereas overall prognostic performance was highest when iron status was modelled using ferritin- and TSAT-based categories, with the combined Ferritin-TSAT framework showing the best discrimination, calibration and fit (C-index 0.76 (0.73–0.78); AIC 1580.07) and identifying an iron-replete subgroup with the lowest adjusted hazard relative to the iron-deficient reference. As detailed in Table 2, Table A1, Table A2, Table A3 and Table A4, the association between iron status and 365-day mortality persisted across progressively adjusted models, although effect estimates varied with adjustment depending on the framework; while clinical covariates behaved consistently (age, shock, malignancy with increased risk; CABG, ACEi/ARB, beta-blocker, IV iron with decreased risk) as associations rather than evidence of causal effects.

The prognostic implications of iron deficiency and hyperferritinemia in hospitalised CHF patients are profound; a study of 1506 CHF patients identified iron deficiency as a strong and independent predictor of mortality (HR 1.42, 95% CI 1.14–1.77, *p* = 0.002) [1]. A stark mortality gradient emerged between iron-deficient and non-iron-deficient cohorts (56.1% vs. 29.4% at one year *p* < 0.001), highlighting iron homeostasis as clinically relevant to cardiac and systemic function. In this cohort, guideline-defined iron deficiency was paradoxically associated with shorter hospital length of stay and lower rates of surgical and ICU admissions, despite substantially higher 1-year mortality compared with non-iron-deficient patients. These utilisation patterns likely reflect differences in case-mix and admission type rather than genuinely lower overall risk: iron-deficient patients were older and predominantly managed medically, with fewer procedures and less ICU exposure, yet they experienced the worst long-term survival. By contrast, the non-iron-deficient group encompassed many hyperferritinemic patients (ferritin ≥ 300 μg/L), a phenotype that in other acute-illness cohorts is strongly linked to systemic inflammation, sepsis, multiorgan dysfunction, greater need for ICU care, and longer ICU and hospital stays. This suggests that hyperferritinemia in hospitalised CHF may mark an acutely inflamed and clinically complex subgroup that requires prolonged hospitalisation, even though their adjusted 365-day mortality in our data was lower than that of the most iron-deficient patients—a comparison that should not be interpreted as hyperferritinemia being “benign,” but rather that, within this high-risk hospitalised population, the most severely iron-deficient patients may be at even higher risk. Importantly, because CRP, hepcidin, and related inflammatory/iron-regulatory markers were not available, we cannot reliably distinguish inflammation- or congestion-driven ferritin elevations from true iron overload in this dataset. Consistent with this pattern, the iron-deficient subgroup had a lower prevalence of AKI, paralleling their lower exposure to ICU care, mechanical ventilation, and vasopressors—factors that are well established to increase AKI risk in critically ill populations. Notably, Kaplan–Meier analyses underscored key distinctions among models (all *p* < 0.001): while guideline-defined classifications exhibited the strongest survival divergence overall (Figure 1), they, alongside TSAT- and ferritin-only category models (Figure 2 and Figure 3), showed limited prognostic utility within the first 30 days. In contrast, the Ferritin-TSAT Category Model (Figure 4) demonstrated sustained late-term yet stronger early-term stratification, distinguishing the iron-replete subgroup from the two higher-risk phenotypes. This showed earlier and sustained curve separation, with hypothesis-generating interpretations that may involve early risks related to impaired oxygen delivery/energetics and late risks related to persistent inflammatory or congestion-associated iron sequestration [2,24]. For reference, the iron deficiency cohort in the Ferritin-TSAT Category Model showed a consistent survival pattern due to its fixed guideline definition across these analyses (Figure 4).

Current guideline criteria, though clinically practical, conflate hyperferritinemic (≥300 μg/L) and normoferritinemic patients under a single “non-iron-deficient” label, obscuring inflammation- or congestion-driven ferritin and iron elevations [10,16,18,25]. Importantly, we recognise limitations of adopting these cutoffs without nuance. Ferritin is an acute-phase reactant and may be elevated despite depleted or unavailable iron in inflammatory states, while TSAT can also shift with changes in transferrin and iron handling during acute illness—potentially leading to misclassification in observational cohorts [16,26]. Consistent with these concerns, bone marrow–validated work suggests that TSAT < 20% is strongly linked to true iron deficiency, and proposes alternative ferritin thresholds (e.g., around ≤128 μg/L when combined with TSAT < 20%) to better distinguish low iron storage from defective utilisation [27,28]. In addition, multiple contemporary analyses have argued for TSAT-centric (or hypoferremia-based) definitions to improve specificity for clinically meaningful iron deficiency in HF [16,26,29]. For these reasons, we intentionally evaluated several competing classification frameworks in parallel (guideline-defined, ferritin-only, TSAT-only, and combined categories), aiming to compare prognostic performance across commonly used and biologically motivated cutoffs rather than presuming a single optimal definition. Additionally, because CRP/hepcidin were unavailable, we could not distinguish inflammation-driven hyperferritinemia from true iron overload at the individual level. TSAT categories (<10%) effectively isolate severe functional deficiency but fail to discern elevated ferritin driven by said inflammation or congestion. Conversely, ferritin categories identify hyperferritinemia but overlook functional deficiency in patients with normal ferritin but critically low TSAT. The Ferritin-TSAT Category Model addresses these gaps by delineating three phenotypes as biochemical strata: (1) true deficiency (low ferritin/TSAT), (2) intermediate-risk, hyperferritinemia, and (3) an iron-replete cohort. Studies show that iron-replete patients often fare better than hyperferritinemic status, substantiating the value of a combined biomarker approach for more precise patient stratification [16,18,24]. Notably, the combined Ferritin-TSAT model isolates a small iron-replete subgroup with the lowest risk, distinct from both the deficient and hyperferritinemic phenotypes (Table A1, Table A2, Table A3 and Table A4). This pattern strengthens the case for moving beyond a binary ‘deficient vs. non-deficient’ framework when risk-stratifying hospitalised CHF patients while recognising that mechanistic attribution requires prospective biomarker-rich studies.

Short-term prognostic results showed an immediate risk carried by severely deficient patients and some hyperferritinemic individuals, whereas long-term mortality confirmed that uncorrected iron dysregulation, be it deficiency or high ferritin, was associated with increased mortality compared to iron-replete [4,30]. By unmasking the prognostic and potential clinical relevance of iron dysregulation subtypes, this model suggests limitations of the current binary paradigm and may inform future clinical guidelines, which currently overlook this maldistribution as a candidate modifiable risk factor. Because these non-binary criteria may help isolate hyperferritinemic individuals, our findings support the hypothesis that future studies could evaluate whether different diagnostic workups and management strategies are warranted across strata; however, our model is intended as a prognostic stratification tool and does not establish a treatment algorithm. Thus, the evaluation of these new categorisations, in conjunction with other pertinent clinical and laboratory measures, may allow for a more individualised approach to preventing adverse outcomes in CHF in future prospective studies. For instance, the ACC/AHA and ESC guidelines for CHF management emphasise iron deficiency correction but lack criteria to address hyperferritinemia or guide differential workups [4,31]. Rather than implying immediate clinical adoption, the Ferritin-TSAT Category Model should be further assessed on more specific retrospective cohorts and then tested as a stratification variable in prospective validation cohorts and interventional studies to evaluate whether management approaches aligned with underlying pathophysiology (e.g., intravenous iron for true deficiency and targeted assessment of congestion/inflammation in hyperferritinemia) improve patient-centred outcomes [16,32]. Although IV iron, ACEi/ARB and beta-blockers were associated with lower hazard after adjustment, these findings remain strictly hypothesis-generating due to potential residual confounding and treatment-selection bias (including the possibility that treated patients received more intensive overall care despite adjusting for variables such as ICU stay).

Recent large-scale datasets have refined how iron deficiency is defined in heart failure and frame our inpatient findings. In the Swedish Heart Failure Registry, compared ESC guideline criteria, IRONMAN trial criteria, TSAT < 20%, and ferritin < 100 μg/L across >20,000 mainly ambulatory patients with HFrEF, HFmrEF, and HFpEF. Iron deficiency was common and consistently linked to worse symptoms and poorer quality of life, but TSAT < 20% and IRONMAN criteria showed the clearest associations with mortality and heart failure hospitalisations, whereas ferritin < 100 μg/L alone carried limited prognostic value [33]. These outpatient data support our focus on TSAT-based and combined Ferritin-TSAT definition as markers of clinically relevant iron deficiency. In contrast, guideline-defined iron deficiency in our hospitalised cohort was associated with 365-day all-cause mortality, implying that identical biochemical thresholds identify a more adverse phenotype once decompensation occurs. The IRONMAN trial extends this prognostic information to treatment response. In 1137 outpatients with chronic heart failure (EF ≤ 45%) and iron deficiency (ferritin < 100 μg/L or TSAT < 20%), intravenous ferric derisomaltose produced larger haemoglobin gains and greater absolute reductions in heart failure hospitalisation or cardiovascular death in patients with lower baseline TSAT and more severe anaemia, whereas those with TSAT ≥ 20% or without anaemia had few events and little benefit [34]. These findings mirror our observation that the most severely iron-deficient inpatients experience the greatest excess mortality and support the hypothesis that this group could derive large absolute benefit if treated effectively—something that must be confirmed in interventional inpatient studies. The EDIFICA acute heart failure cohort complements these results by dissecting ferritin as a marker of both iron status and inflammation. In 526 patients, higher admission ferritin independently predicted a higher 180-day risk of heart failure hospitalisation or cardiovascular death, whereas low ferritin and guideline-defined iron deficiency were not robust markers of risk; at discharge, defective iron utilisation or guideline-defined iron deficiency were paradoxically associated with lower risk than apparent iron repletion [35]. EDIFICA therefore concludes that hyperferritinemia in acute heart failure frequently reflects an inflammatory “hyperferritinemic” phenotype with restricted iron bioavailability and that reliance on ferritin thresholds alone risks undertreating this subgroup: an interpretation that aligns with our separation of a hyperferritinemic inpatient phenotype, thereby supporting a more nuanced, phenotype-oriented approach than a simple binary “deficient versus non-deficient” framework, and our emphasis on TSAT and combined Ferritin-TSAT frameworks for risk stratification.

Early iron parameter assessment (within 24 h of admission) could identify high-risk patients prone to early decompensation, as both deficiency and hyperferritinemia correlate with rapid clinical decline. Multivariate analyses were consistent with an independent association between iron dysregulation and mortality, unaffected by covariates such as shock, malignancy, or ICU admission. Importantly, standard CHF therapies (beta-blockers, ACE inhibitors/ARBs) may interact clinically with iron status optimisation, but our data cannot infer causal interaction effects. In patients with low ferritin or TSAT, especially below the accepted guideline thresholds, intravenous iron therapy has shown clinical benefit in prior trials (e.g., FAIR-HF, CONFIRM-HF, AFFIRM-AHF, IRONMAN). In our cohort, intravenous iron exposure was associated with lower adjusted hazard, but this should not be interpreted as evidence of causal benefit because of confounding by indication and treatment-selection bias. Rather, our findings support the hypothesis that early, phenotype-guided trials (e.g., enrolling patients with very low TSAT and/or low ferritin) could test whether early repletion reduces short-term events and attenuates one-year risk, whereas hyperferritinemic patients—often misclassified as “non-deficient”—may require different evaluation pathways that should be defined and tested prospectively, likely integrating inflammatory and congestive markers alongside prespecified diagnostic and management strategies to be tested.

### Limitations

Several limitations warrant caution in interpreting these findings. The dataset is based on a retrospective review of records from a single centre, Beth Israel Deaconess Medical Centre, which may limit generalisability to other institutions or healthcare settings with different patient demographics and clinical practices. All-cause mortality was the only outcome that could be consistently ascertained, as death can be verified after discharge through available mortality sources. As with most retrospective analyses, causal inferences cannot be definitively established, and observed associations between iron parameters and outcomes may be influenced by unmeasured confounders. Moreover, residual confounding related to treatment-selection (confounding by indication) is possibly present: the apparent protective associations of IV iron, ACEi/ARB therapy, and beta-blockers may reflect underlying clinical stability, contraindication patterns, blood pressure/renal function constraints, or clinician selection rather than true treatment benefit.

During dataset creation, some inflammatory markers were not routinely measured, making it difficult to distinguish between true iron overload (e.g., hemochromatosis) from inflammation-driven ferritin elevations due to limited aetiologic coding and inflammatory biomarkers; future work incorporating CRP, hepcidin, and sTfR is indeed warranted. Inflammation was partially assessed, as neutrophil count was initially measured, but did not materially change estimates. Addressing this gap in future investigations could provide a clearer understanding of inflammatory mechanisms in iron dysregulation and potential mortality risk in heart failure. Additionally, requiring iron indices to be measured within the first 24 h necessitated excluding patients without early iron testing, which may introduce selection bias by enriching the cohort for sicker patients, those undergoing more intensive evaluation, or those managed in settings where iron assessment is more routinely pursued.

The need for prospective validation of these findings should be emphasised, stratifying patients into nuanced Ferritin-TSAT categories and testing personalised iron therapies. The analysis includes large numbers of patients and uses multiple adjustment models that incorporate age, shock, malignancy, renal function, ejection fraction, and comedications, supporting that iron status may be associated with mortality independent of measured covariates. By comparing guideline-defined iron deficiency, TSAT-based regression, ferritin-based categories, and a combined Ferritin-TSAT model, the study gives a comprehensive perspective on how best to capture diverse risk groups in CHF. However, incorporating dedicated inflammatory biomarkers would clarify whether hyperferritinemia is truly synonymous with iron repletion or driven by an acute-phase response that blocks normal iron utilisation. Tracking iron indices beyond a single time point may also show how dynamic changes in iron status correlate with readmissions or advanced interventions for CHF. Overall, these findings should be considered hypothesis-generating only and should not be interpreted as demonstrating treatment efficacy as causal claims require randomised evidence. Accordingly, any observed associations between therapies (including IV iron) and outcomes in this dataset should be framed as signals for further study rather than evidence of benefit.

## 5. Conclusions

This study evaluated iron status—both deficient and excessively high—as a set of prognostic markers of CHF outcomes in a retrospective cohort. While guideline-defined iron deficiency remains a strong, practical predictor of early and longer-term mortality, a significant proportion of patients with ferritin ≥ 300 μg/L may also exhibit poor survival trajectories. However, because CRP, hepcidin, and related inflammatory/iron-regulatory biomarkers were unavailable, we could not reliably distinguish inflammation- or congestion-driven ferritin elevations from true iron overload at the individual level. We hypothesize that non-binary models offer finer discrimination between true deficiency and hyperferritinemia, supporting improved risk stratification and the generation of testable hypotheses about underlying mechanisms. Accordingly, future studies could evaluate whether ferritin-TSAT defined subgroups differ in diagnostic needs or responses to therapy; however, our findings do not establish a treatment algorithm or treatment efficacy, which remain the domain of prospective and randomised trials. By identifying iron dysregulation early in the course of hospitalisation, clinicians may consider early risk stratification using iron indices, although we emphasise that additional observational studies with key missing biomarkers and subsequent interventional and randomised trials are required before any changes to diagnostic pathways or treatment protocols can be recommended. To conclude, our 3-class Ferritin-TSAT model should be considered a prognostic tool that requires further validation with different cutoffs and more biomarker-rich covariates, particularly inflammatory and iron-regulatory measures.

## Figures and Tables

**Table 1:** Baseline patient demographics, laboratory values, comorbidities, medications, procedures, and mortality outcomes stratified by iron deficiency status. Iron deficiency defined as ferritin < 100 μg/L or ferritin 100–299 μg/L with TSAT < 20% (guideline definition). Continuous variables are median (IQR).

Variable	Non-Iron Deficiency (n = 1220)	Iron Deficiency (n = 619)	*p*-Value
**Demographics**			
Age	72.00 (18.00)	77.00 (18.00)	<0.001
Sex (female)	431 (35.3%)	307 (49.6%)	<0.001
**Race**			
White	812 (66.6%)	435 (70.3%)	
Asian	33 (2.7%)	8 (1.3%)	
Hispanic	59 (4.8%)	29 (4.7%)	
Black	23 (1.9%)	9 (1.5%)	
Mixed/Other	293 (24.0%)	138 (22.3%)	0.241
**Marital status**			
Married	562 (46.1%)	261 (42.2%)	
Widowed	173 (14.2%)	132 (21.3%)	
Divorced	96 (7.9%)	56 (9.0%)	
Single	309 (25.3%)	140 (22.6%)	
Other	80 (6.6%)	30 (4.9%)	0.001
**Hospitalisation**			
Surgical admission	138 (11.3%)	28 (4.5%)	<0.001
Hospital LOS	8.94 (8.58)	6.98 (7.80)	<0.001
ICU stay	698 (57.2%)	211 (34.1%)	<0.001
ICU LOS	3.16 (4.80)	2.36 (3.4)	<0.001
**Laboratory**			
Troponin	0.11 (0.32)	0.07 (0.16)	<0.001
Ferritin	392.50 (515.75)	71.00 (74.00)	<0.001
Iron	45.00 (45.00)	31.00 (22.00)	<0.001
TIBC	234.00 (89.00)	332.00 (110.50)	<0.001
Transferrin	180.00 (68.00)	255.00 (84.50)	<0.001
TSAT	19.91 (15.72)	9.64 (7.33)	<0.001
Haemoglobin	10.10 (3.50)	9.20 (2.70)	<0.001
Hematocrit	31.17 (10.39)	29.70 (8.14)	<0.001
RBC	3.43 (1.18)	3.46 (1.00)	0.288
RDW	15.30 (2.90)	16.60 (3.53)	<0.001
Platelets	189.00 (112.00)	211.00 (110.00)	<0.001
Glucose	143.00 (83.00)	139.00 (84.00)	0.187
Creatinine	1.60 (1.60)	1.50 (1.10)	<0.001
eGFR	44.93 (49.16)	46.64 (41.99)	0.004
Ejection fraction	40.00 (25.00)	40.00 (25.00)	0.635
CKD-EPI Group			<0.001
1	151 (12.4%)	79 (12.8%)	
2	260 (21.3%)	141 (22.8%)	
3	400 (32.8%)	250 (40.4%)	
4	241 (19.8%)	120 (19.4%)	
5	168 (13.8%)	29 (4.7%)	
Ejection fraction Group			0.447
<40	442 (36.2%)	241 (38.9%)	
41–49	233 (19.1%)	107 (17.3%)	
>50	545 (44.7%)	271 (43.8%)	
**Comorbidities**			
STEMI	32 (2.6%)	4 (0.6%)	0.007
NSTEMI	254 (20.8%)	84 (13.6%)	<0.001
Cardiac arrest	39 (3.2%)	8 (1.3%)	0.022
Valvopathies	434 (35.6%)	304 (49.1%)	<0.001
Mitral valve disease	249 (20.4%)	168 (27.1%)	0.001
Aortic valve disease	174 (14.3%)	118 (19.1%)	0.009
Atrial fibrillation	559 (45.8%)	330 (53.3%)	0.003
Hypertension	989 (81.1%)	522 (84.3%)	0.096
Hyperlipidemia	850 (69.7%)	440 (71.1%)	0.568
Peripheral vascular disease	335 (27.5%)	175 (28.3%)	0.755
Diabetes	591 (48.4%)	319 (51.5%)	0.229
Chronic kidney failure	631 (51.7%)	322 (52.0%)	0.943
Acute kidney failure	315 (25.8%)	88 (14.2%)	<0.001
Mild liver disease	124 (10.2%)	58 (9.4%)	0.648
Severe liver disease	43 (3.5%)	17 (2.7%)	0.454
Malignant cancer	121 (9.9%)	49 (7.9%)	0.188
Obesity	182 (14.9%)	129 (20.8%)	0.002
Alcoholism	225 (18.4%)	73 (11.8%)	<0.001
Shock	309 (25.3%)	90 (14.5%)	<0.001
Prior coronary stent	175 (14.3%)	112 (18.1%)	0.036
Prior bypass graft	179 (14.7%)	112 (18.1%)	0.067
History of major CV event *	195 (16.0%)	124 (20.0%)	0.036
**Medications**			
Antiplatelets	932 (76.4%)	445 (71.9%)	0.041
Aspirin	920 (75.4%)	430 (69.5%)	0.008
P2Y12 Inhibitors	298 (24.4%)	113 (18.3%)	0.003
Anticoagulants	489 (40.1%)	318 (51.4%)	<0.001
Vasopressors	440 (36.1%)	129 (20.8%)	<0.001
ACEi	397 (32.5%)	178 (28.8%)	0.109
ARB	192 (15.7%)	106 (17.1%)	0.487
Beta blockers	1008 (82.6%)	499 (80.6%)	0.320
CCB	265 (21.7%)	113 (18.3%)	0.094
Statins	911 (74.7%)	466 (75.3%)	0.819
Iron supplement	-	314 (50.7%)	-
Iron supplement (IV)	-	191 (30.9%)	-
Iron supplement (PO)	-	159 (25.7%)	-
**Procedures and Interventions**			
CABG	66 (5.4%)	11 (1.8%)	<0.001
PCI	95 (7.8%)	22 (3.6%)	0.001
IABP	30 (2.5%)	10 (1.6%)	0.316
Mechanical support	49 (4.0%)	15 (2.4%)	0.104
Pacemaker	130 (10.7%)	237 (38.3%)	<0.001
Implantable defibrillator	143 (11.7%)	210 (33.9%)	<0.001
Valve surgery	61 (5.0%)	24 (3.9%)	0.334
Dialysis	120 (9.8%)	20 (3.2%)	<0.001
Ventilation	501 (41.1%)	175 (28.3%)	<0.001
**Mortality**			
14-day Mortality	93 (7.6%)	52 (8.4%)	0.622
28-day Mortality	149 (12.2%)	81 (13.1%)	0.646
90-day Mortality	231 (18.9%)	178 (28.8%)	<0.001
180-day Mortality	281 (23.0%)	243 (39.3%)	<0.001
365-day Mortality	359 (29.4%)	347 (56.1%)	<0.001
Hospital Mortality	108 (8.9%)	51 (8.2%)	0.723

* History of CV outcomes constituted. Abbreviations: ACEi, angiotensin-converting enzyme inhibitor; ARB, angiotensin II receptor blocker; CABG, coronary artery bypass grafting; CCB, calcium channel blocker; CKD-EPI, Chronic Kidney Disease Epidemiology Collaboration; CV, cardiovascular; eGFR, estimated glomerular filtration rate; IABP, intra-aortic balloon pump; ICU, intensive care unit; IV, intravenous; LOS, length of stay; NSTEMI, non-ST-elevation myocardial infarction; PCI, percutaneous coronary intervention; PO, per os (oral); RBC, red blood cell count; RDW, red cell distribution width; STEMI, ST-elevation myocardial infarction; TIBC, total iron-binding capacity; TSAT, transferrin saturation.

**Table 2:** Hazard ratios and confidence intervals for iron deficiency regressions across multiple crude and adjusted multivariate and univariate models.

Iron Variable Model	Model 1	Model 2	Model 3	Univariate
Guideline-defined Model	3.350 (95% CI 2.730–4.110), *p* < 0.001	3.787 (95% CI 3.053–4.697), *p* < 0.001	4.356 (95% CI 3.349–5.338), *p* < 0.001	2.48 (95% CI 2.09–2.94), *p* < 0.001
TSAT Category Model	Tertile 2: 0.753 (95% CI 0.610–0.930), *p* = 0.009	Tertile 2: 0.780 (95% CI 0.626–0.971), *p* = 0.026	Tertile 2: 0.804 (95% CI 0.644–1.003), *p* = 0.053	Tertile 2: 0.72 (95% CI 0.613–0.849), *p* < 0.001
	Tertile 3: 0.609 (95% CI 0.484–0.766), *p* < 0.001	Tertile 3: 0.625 (95% CI 0.492–0.794), *p* < 0.001	Tertile 3: 0.632 (95% CI 0.494–0.810), *p* < 0.001	Tertile 3: 0.584 (95% CI 0.424–0.783), *p* < 0.001
Ferritin Category Model	Tertile 2: 0.671 (95% CI 0.544–0.827), *p* < 0.001	Tertile 2: 0.649 (95% CI 0.523–0.804), *p* < 0.001	Tertile 2: 0.670 (95% CI 0.538–0.833), *p* < 0.001	Tertile 2: 0.662 (95% CI 0.507–0.813), *p* < 0.001
	Tertile 3: 0.400 (95% CI 0.313–0.511), *p* < 0.001	Tertile 3: 0.362 (95% CI 0.280–0.468), *p* < 0.001	Tertile 3: 0.308 (95% CI 0.234–0.404), *p* < 0.001	Tertile 3: 0.388 (95% CI 0.304–0.574), *p* < 0.001
Ferritin-TSAT Category Model	Tertile 2: 0.337 (95% CI 0.228–0.496), *p* < 0.001	Tertile 2: 0.338 (95% CI 0.228–0.501), *p* < 0.001	Tertile 2: 0.356 (95% CI 0.238–0.530), *p* < 0.001	Tertile 2: 0.306 (95% CI 0.228–0.496), *p* < 0.001
	Tertile 3: 0.498 (95% CI 0.328–0.517), *p* < 0.001	Tertile 3: 0.551 (95% CI 0.389–0.622), *p* = 0.001	Tertile 3: 0.495 (95% CI 0.380–0.512), *p* < 0.001	Tertile 3: 0.517 (95% CI 0.315–0.583), *p* < 0.001

Tertile category models encompass multiple values as they categorise data into three distinct groups and show reference categories: TSAT < 10%; ferritin < 100 μg/L; combined guideline-defined iron deficiency. HR < 1 indicates lower hazard vs. reference, not absolute benefit. categories 2 and 3 HRs are calculated in relation to Tertile 1, with each Tertile referencing categories as seen in Figure 1, Figure 2, Figure 3 and Figure 4 and defined with each category model. Reported HRs are from Model 3 (fully adjusted) unless specified. Table A1, Table A2, Table A3 and Table A4 show full multivariate models. Abbreviations: CI, confidence interval; TSAT, transferrin saturation.

**Table 3:** Subgroup analyses of 365-day mortality by iron status across prespecified strata. Values are hazard ratios (HRs) with 95% confidence intervals from fully adjusted Cox models *.

Subgroup	Level	Guideline ID: HR (95% CI) *	p-Interaction †	Ferritin-TSAT: Iron-Replete (95% CI) §	Ferritin-TSAT: Hyperferritinemia (95% CI) §	p-Interaction ‡
Age	<65	3.95 (3.02–5.16)	0.38	0.34 (0.21–0.54)	0.54 (0.39–0.74)	0.44
	≥65	4.52 (3.34–6.11)		0.41 (0.28–0.59)	0.50 (0.38–0.66)	
Sex	Female	4.68 (3.36–6.51)	0.62	0.36 (0.23–0.57)	0.52 (0.38–0.70)	0.71
	Male	4.22 (3.21–5.56)		0.39 (0.26–0.58)	0.49 (0.37–0.64)	
EF category	<40%	4.85 (3.45–6.82)	0.29	0.34 (0.23–0.51)	0.48 (0.36–0.65)	0.33
	40–49%	3.72 (2.11–6.56)		0.58 (0.30–1.11)	0.70 (0.41–1.18)	
	≥50%	3.98 (2.95–5.36)		0.39 (0.26–0.59)	0.51 (0.37–0.70)	

* Guideline ID compares ID vs. non-ID within each stratum; § Ferritin-TSAT reference = True ID (ferritin < 100 or 100–299 with TSAT < 20%); † p-interaction (1-df) for Guideline ID × subgroup; ‡ p-interaction (2-df, global) for Ferritin-TSAT × subgroup. P-interaction was calculated using Wald test. Abbreviations: CI, confidence interval; df, degrees of freedom; EF, ejection fraction; HR, hazard ratio; ID, iron deficiency; TSAT, transferrin saturation.

## Data Availability

The data that support the findings of this study are derived from the MIMIC-IV database, which is publicly available at https://physionet.org/content/mimiciv/2.2/ (accessed on 15 August 2025) for credentialed researchers who complete the required data use agreement.

## Abbreviations

The following abbreviations are used in this manuscript:

ACC	American College of Cardiology
ACEi	Angiotensin-converting enzyme inhibitor
AHA	American Heart Association
AKI	Acute kidney injury
ARB	Angiotensin II receptor blocker
ARNI	Angiotensin receptor–neprilysin inhibitor
AIC	Akaike information criterion
Brier	Brier score
CABG	Coronary artery bypass grafting
CCB	Calcium channel blocker
CHF	Congestive heart failure
CI	Confidence interval
CKD	Chronic kidney disease
CKD-EPI	Chronic Kidney Disease Epidemiology Collaboration
CRP	C-reactive protein
CV	Cardiovascular
EHR	Electronic health record
EF	Ejection fraction
eGFR	Estimated glomerular filtration rate
EMR	Electronic medical record
ESC	European Society of Cardiology
HF	Heart failure
HFmrEF	Heart failure with mildly reduced ejection fraction
HFpEF	Heart failure with preserved ejection fraction
HFrEF	Heart failure with reduced ejection fraction
HFA	Heart Failure Association (of the ESC)
HFSA	Heart Failure Society of America
HR	Hazard ratio
IABP	Intra-aortic balloon pump
ICD	International Classification of Diseases
ICU	Intensive care unit
ID	Iron deficiency
IQR	Interquartile range
IV	Intravenous
KDIGO	Kidney Disease: Improving Global Outcomes
LOS	Length of stay
LVEF	Left ventricular ejection fraction
MCAR	Missing completely at random
MIMIC-IV	Medical Information Mart for Intensive Care IV
MRA	Mineralocorticoid receptor antagonist
NSTEMI	Non-ST-elevation myocardial infarction
PCI	Percutaneous coronary intervention
PO	Per os (oral)
Q–Q	Quantile–quantile
RBC	Red blood cell count
RDW	Red cell distribution width
SGLT2i	Sodium–glucose cotransporter-2 inhibitor
SD	Standard deviation
sTfR	Soluble transferrin receptor
STEMI	ST-elevation myocardial infarction
TIBC	Total iron-binding capacity
TSAT	Transferrin saturation

## Appendix A

### Appendix A.1

**Table A1:** Fully adjusted hazard ratios and confidence intervals for covariates with the Guideline-defined Model.

	Guideline-Defined Model (4.356, 95% CI 3.349–5.338, *p* < 0.001)	
	HR	CI
Age	1.036	95% CI 1.03–1.05, *p* < 0.001
Sex	1.105	95% CI 0.92–1.34, *p* = 0.299
Mildly-Reduced Ejection Fraction	0.788	95% CI 0.51–1.24, *p* = 0.299
Preserved Ejection Fraction	0.887	95% CI 0.74–1.08, *p* = 0.212
ICU Stay	1.480	95% CI 1.19–1.86, *p* = 0.001
Acute Coronary Artery Disease	2.126	95% CI 1.18–3.84, *p* = 0.012
Hypertension	1.130	95% CI 0.87–1.48, *p* = 0.374
Shock	1.978	95% CI 1.53–2.56, *p* < 0.001
Diabetes	1.496	95% CI 1.24–1.81, *p* < 0.001
Malignant Cancer	1.501	95% CI 1.14–1.98, *p* = 0.004
Haemoglobin (log10) (g/dL)	0.687	95% CI 0.25–1.95, *p* = 0.48
eGFR (log10) (mL/min/1.73 m^2^)	0.738	95% CI 0.52–1.07, *p* = 0.101
CABG	0.314	95% CI 0.14–0.72, *p* = 0.005
PCI	0.653	95% CI 0.40–1.08, *p* = 0.097
ACEi/ARB Use	0.684	95% CI 0.56–0.84, *p* < 0.001
Dialysis Status	0.471	95% CI 0.33–0.68, *p* < 0.001
IV Iron Supplementation	0.571	95% CI 0.45–0.73, *p* < 0.001
Beta-Blocker Use	0.659	95% CI 0.53–0.82, *p* < 0.001
Calcium Channel Blocker Use	0.673	95% CI 0.53–0.87, *p* = 0.002

Abbreviations: ICU: Intensive care unit; eGFR: estimated glomerular filtration rate; CABG: coronary artery bypass graft; PCI: percutaneous coronary intervention; ACEi/ARB: Angiotensin converting enzyme inhibitor/Angiotensin II receptor blockers; IV: intravenous. Harrell’s C-index 0.72 (0.70–0.74); AIC 1650.21; Brier 0.20.

### Appendix A.2

**Table A2:** Fully adjusted hazard ratios and confidence intervals for covariates with the TSAT Category Model.

	TSAT Category Model (Tertile 2: 0.804, 95% CI 0.644–1.003, *p* = 0.053 Tertile 3: 0.632, 95% CI 0.494–0.810, *p* < 0.001)	
	HR	CI
Age	1.045	95% CI 1.04–1.05, *p* < 0.001
Sex	1.275	95% CI 0.99–1.44, *p* = 0.078
Mildly-Reduced Ejection Fraction	0.699	95% CI 0.50–1.23, *p* = 0.278
Preserved Ejection Fraction	0.775	95% CI 0.67–0.99, *p* = 0.031
ICU Stay	1.247	95% CI 1.15–1.80, *p* = 0.002
Acute Coronary Artery Disease	1.579	95% CI 0.85–2.75, *p* = 0.159
Hypertension	1.179	95% CI 0.89–1.52, *p* = 0.280
Shock	1.836	95% CI 1.40–2.34, *p* < 0.001
Diabetes	1.560	95% CI 1.25–1.82, *p* < 0.001
Malignant Cancer	1.398	95% CI 1.12–1.95, *p* = 0.006
Haemoglobin (log10) (g/dL)	0.506	95% CI 0.21–1.72, *p* = 0.340
eGFR (log10) (mL/min/1.73 m^2^)	1.046	95% CI 0.55–1.14, *p* = 0.209
CABG	0.255	95% CI 0.11–0.53, *p* < 0.001
PCI	0.685	95% CI 0.37–1.00, *p* = 0.050
ACEi/ARB Use	0.685	95% CI 0.54–0.82, *p* < 0.001
Dialysis Status	0.564	95% CI 0.34–0.69, *p* < 0.001
IV Iron Supplementation	0.825	95% CI 0.54–0.87, *p* = 0.002
Beta-Blocker Use	0.681	95% CI 0.53–0.82, *p* < 0.001
Calcium Channel Blocker Use	0.684	95% CI 0.53–0.87, *p* = 0.002

Abbreviations: ICU: Intensive care unit; eGFR: estimated glomerular filtration rate; CABG: coronary artery bypass graft; PCI: percutaneous coronary intervention; ACEi/ARB: Angiotensin converting enzyme inhibitor/Angiotensin II receptor blockers; IV: intravenous. Harrell’s C-index 0.71 (0.68–0.74); AIC 1680.84; Brier 0.23.

### Appendix A.3

**Table A3:** Fully adjusted hazard ratios and confidence intervals for covariates with the Ferritin Category Model.

	Ferritin Category Model (Tertile 2: 0.670, 95% CI 0.538–0.833, *p* < 0.001 Tertile 3: 0.308, 95% CI 0.234–0.404, *p* < 0.001)	
	HR	CI
Age	1.040	95% CI 1.04–1.05, *p* < 0.001
Sex	1.186	95% CI 0.99–1.44, *p* = 0.078
Mildly-Reduced Ejection Fraction	0.780	95% CI 0.50–1.23, *p* = 0.278
Preserved Ejection Fraction	0.811	95% CI 0.67–0.99, *p* = 0.031
ICU Stay	1.433	95% CI 1.15–1.80, *p* = 0.002
Acute Coronary Artery Disease	1.526	95% CI 0.85–2.75, *p* = 0.159
Hypertension	1.160	95% CI 0.89–1.52, *p* = 0.280
Shock	1.805	95% CI 1.40–2.34, *p* < 0.001
Diabetes	1.507	95% CI 1.25–1.82, *p* < 0.001
Malignant Cancer	1.472	95% CI 1.12–1.95, *p* = 0.006
Haemoglobin (log10) (g/dL)	0.599	95% CI 0.21–1.72, *p* = 0.340
eGFR (log10) (mL/min/1.73 m^2^)	0.792	95% CI 0.55–1.14, *p* = 0.209
CABG	0.231	95% CI 0.11–0.53, *p* < 0.001
PCI	0.604	95% CI 0.37–1.00, *p* = 0.050
ACEi/ARB Use	0.661	95% CI 0.54–0.82, *p* < 0.001
Dialysis Status	0.477	95% CI 0.34–0.69, *p* < 0.001
IV Iron Supplementation	0.681	95% CI 0.54–0.87, *p* = 0.002
Beta-Blocker Use	0.658	95% CI 0.53–0.82, *p* < 0.001
Calcium Channel Blocker Use	0.674	95% CI 0.53–0.87, *p* = 0.002

Abbreviations: ICU: Intensive care unit; eGFR: estimated glomerular filtration rate; CABG: coronary artery bypass graft; PCI: percutaneous coronary intervention; ACEi/ARB: Angiotensin converting enzyme inhibitor/Angiotensin II receptor blockers; IV: intravenous. Harrell’s C-index 0.73 (0.70–0.75); AIC 1610.19; Brier 0.18.

### Appendix A.4

**Table A4:** Fully adjusted hazard ratios and confidence intervals for covariates with the Ferritin-TSAT Category Model.

	Ferritin-TSAT Category Model (Tertile 2: 0.356, 95% CI 0.238–0.530, *p* < 0.001 Tertile 3: 0.495, 95% CI 0.380–0.512, *p* < 0.001)	
	HR	CI
Age	1.039	95% CI 1.03–1.05, *p* < 0.001
Sex	1.191	95% CI 0.99–1.44, *p* = 0.070
Mildly-Reduced Ejection Fraction	0.774	95% CI 0.50–1.22, *p* = 0.262
Preserved Ejection Fraction	0.812	95% CI 0.68–0.99, *p* = 0.031
ICU Stay	1.437	95% CI 1.15–1.80, *p* = 0.002
Acute Coronary Artery Disease	1.922	95% CI 1.06–3.50, *p* = 0.032
Hypertension	1.163	95% CI 0.89–1.53, *p* = 0.271
Shock	1.847	95% CI 1.43–2.40, *p* < 0.001
Diabetes	1.488	95% CI 1.24–1.80, *p* < 0.001
Malignant Cancer	1.474	95% CI 1.12–1.95, *p* = 0.006
Haemoglobin (log10) (g/dL)	0.599	95% CI 0.21–1.72, *p* = 0.340
eGFR (log10) (mL/min/1.73 m^2^)	0.824	95% CI 0.58–1.19, *p* = 0.295
CABG	0.224	95% CI 0.10–0.51, *p* < 0.001
PCI	0.646	95% CI 0.39–1.08, *p* = 0.090
ACEi/ARB Use	0.683	95% CI 0.56–0.84, *p* < 0.001
Dialysis Status	0.505	95% CI 0.36–0.73, *p* < 0.001
IV Iron Supplementation	0.670	95% CI 0.53–0.86, *p* = 0.001
Beta-Blocker Use	0.665	95% CI 0.54–0.83, *p* < 0.001
Calcium Channel Blocker Use	0.682	95% CI 0.53–0.88, *p* = 0.003

Abbreviations: ICU: Intensive care unit; eGFR: estimated glomerular filtration rate; CABG: coronary artery bypass graft; PCI: percutaneous coronary intervention; ACEi/ARB: Angiotensin converting enzyme inhibitor/Angiotensin II receptor blockers; IV: intravenous. Harrell’s C-index 0.76 (0.73–0.78); AIC 1580.07; Brier 0.16.
